# Characterization of the complete plastome of *Atriplex centralasiatica* (Chenopodiaceae), an annual halophytic herb

**DOI:** 10.1080/23802359.2019.1638329

**Published:** 2019-07-13

**Authors:** Xue-Jie Zhang, Ning Wang, Luo-Yan Zhang, Shou-Jin Fan, Xiao-Jian Qu

**Affiliations:** Key Lab of Plant Stress Research, College of Life Sciences, Shandong Normal University, Ji’nan, Shandong, China

**Keywords:** *Atriplex centralasiatica*, plastome, phylogenomics

## Abstract

*Atriplex centralasiatica*, an annual halophytic herb, is one of the most important Chinese herbal medicines, forages and indicator plants for saline-alkali soil. In this study, we report the complete plastome of *A. centralasiatica*. The plastome was 152,237 bp in length and comprises a large single-copy region (83,721 bp), a small single-copy region (18,096 bp), and a pair of inverted repeats (25,210 bp). It encodes 113 unique genes, including 79 protein-coding genes (PCGs), 30 tRNAs and 4 rRNAs. The overall GC content of this plastome was 37.3%. Phylogenomic analysis based on 21 plastomes revealed that *A. centralasiatica* was closely related to the genus *Chenopodium*.

*Atriplex centralasiatica*, an annual halophytic herb, is one of the most important indicator plants in the saline-alkali region of China (Zhang et al. 2010; Yuan et al. 2016). This species, together with other halophytes as *Suaeda salsa* and *Thellungiella halophila*, play an important role in investigating the physiological mechanisms of salt tolerance in halophytes (Sui et al. 2010; Han et al. 2011; Guo, Jia et al. 2012; Guo, Wang et al. 2012; Han et al. 2012; Li, Liu et al. 2012; Li, Pang et al. 2012; Xu et al. 2013; Cheng et al. 2014; Feng et al. 2014; Sui and Han 2014; Guo et al. 2015; Wang et al. 2017; Cui et al. 2018; Guo et al. 2018). Photosynthesis in Chenopodiaceae C4 plants is considered to help improve the adaptability of C4 plants in saline environments (Qi et al. 2010; Guo, Wang et al. 2012). *Atriplex centralasiatica* is one of the representative C4 halophytes in Chenopodiaceae. Yet, its photosynthetic characteristics under salt stress have seldom been investigated. In this study, we reported the plastome of *A. centralasiatica*, the first one in genus *Atriplex*, which would provide fundamental genetic resource for studying this important species.

Fresh leaves of *A. centralasiatica* were collected from the Yellow River Delta (Shandong, China; 37°29'N, 118°43'E). Voucher specimen (XLC47) was deposited at College of Life Sciences, Shandong Normal University. Total genomic DNA was extracted by the modified CTAB method described in Wang et al. (2013). Due to limited fresh sample, the plastid DNA was not directly extracted (Liu et al. 2017). The total genomic DNA was used for library preparation and paired-end (PE) sequencing by the Illumina MiSeq instrument at Novogene (Beijing, China). The plastome was assembled using OGA-Organelle Genome Assembler (https://github.com/quxiaojian/OGA; Qu 2019). Annotation was performed with PGA-Plastid Genome Annotator (Qu et al. 2019), coupled with manual correction using Geneious v8.0.2 (https://www.geneious.com). To determine the phylogenetic placement of *A. centralasiatica*, a maximum likelihood (ML) tree was reconstructed using RAxML v8.2.10 (Stamatakis 2014), including tree robustness assessment using 1000 rapid bootstrap replicates with the GTRGAMMA substitution model, based on the alignment of 79 shared PCGs using MAFFT v7.313 (Katoh and Standley 2013).

The complete plastome of *A. centralasiatica* (GenBank accession number: MK867774) was 152,237 bp in length and comprises a large single-copy region (LSC: 83,721 bp), a small single-copy region (SSC: 18,096 bp), and a pair of inverted repeats (IR: 25,210bp). The overall GC content was 37.3%. A total of 113 unique genes were annotated in this plastome, including 79 protein-coding genes (PCGs), 30 tRNAs and 4 rRNAs. Among them, eleven PCGs and six tRNAs contained introns, in which nine PCGs and six tRNAs contained one intron and two PCGs contained two introns. There were 18 duplicated genes in the IR. The ML phylogenetic tree showed that *A. centralasiatica* was closely related to the genus *Chenopodium* (Figure 1).

**Figure 1.**
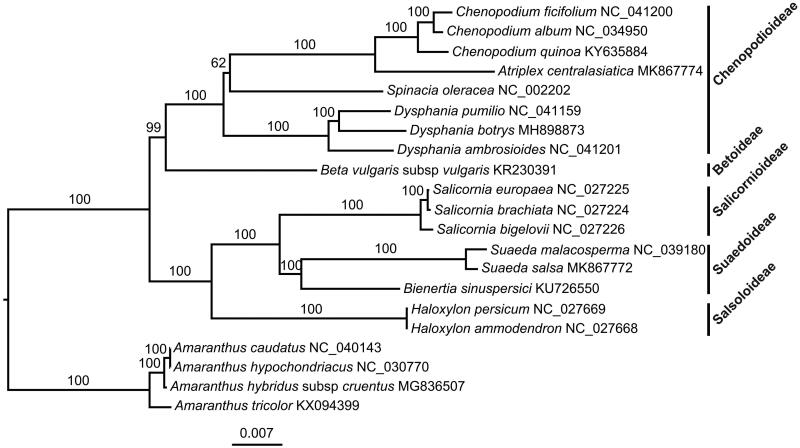
A maximum likelihood (ML) tree inferred from 79 plastome genes is shown. Four *Amaranthus* species from Amaranthaceae are used as outgroup. The numbers on branches are bootstrap support values.
